# Corrigendum to “Protective Effects of Soy Oligopeptides in Ultraviolet B-Induced Acute Photodamage of Human Skin”

**DOI:** 10.1155/2018/3871280

**Published:** 2018-01-10

**Authors:** Bing-rong Zhou, Li-wen Ma, Juan Liu, Jia-an Zhang, Yang Xu, Di Wu, Felicia Permatasari, Dan Luo

**Affiliations:** ^1^Department of Dermatology, The First Affiliated Hospital of Nanjing Medical University, Nanjing 210029, China; ^2^Drum Tower Hospital, Medical School of Nanjing University, Nanjing, China

In the article titled “Protective Effects of Soy Oligopeptides in Ultraviolet B-Induced Acute Photodamage of Human Skin” [1], the affiliation of the second author was missing. The corrected authors' list and affiliations are shown above.
